# Improving health-related fitness in children: the fit-4-Fun randomized controlled trial study protocol

**DOI:** 10.1186/1471-2458-11-902

**Published:** 2011-12-05

**Authors:** Narelle Eather, Philip J Morgan, David R Lubans

**Affiliations:** 1 Priority Research Centre in Physical Activity and Nutrition, School of Education, University of Newcastle, Callaghan Campus, Newcastle, Australia; 2 University of Newcastle, Newcastle, Australia

## Abstract

**Background:**

Declining levels of physical fitness in children are linked to an increased risk of developing poor physical and mental health. Physical activity programs for children that involve regular high intensity physical activity, along with muscle and bone strengthening activities, have been identified by the World Health Organisation as a key strategy to reduce the escalating burden of ill health caused by non-communicable diseases. This paper reports the rationale and methods for a school-based intervention designed to improve physical fitness and physical activity levels of Grades 5 and 6 primary school children.

**Methods/Design:**

Fit-4-Fun is an 8-week multi-component school-based health-related fitness education intervention and will be evaluated using a group randomized controlled trial. Primary schools from the Hunter Region in NSW, Australia, will be invited to participate in the program in 2011 with a target sample size of 128 primary schools children (age 10-13). The Fit-4-Fun program is theoretically grounded and will be implemented applying the Health Promoting Schools framework. Students will participate in weekly curriculum-based health and physical education lessons, daily break-time physical activities during recess and lunch, and will complete an 8-week (3 × per week) home activity program with their parents and/or family members. A battery of six health-related fitness assessments, four days of pedometery-assessed physical activity and a questionnaire, will be administered at baseline, immediate post-intervention (2-months) and at 6-months (from baseline) to determine intervention effects. Details of the methodological aspects of recruitment, inclusion criteria, randomization, intervention program, assessments, process evaluation and statistical analyses are described.

**Discussion:**

The Fit-4-Fun program is an innovative school-based intervention targeting fitness improvements in primary school children. The program will involve a range of evidence-based behaviour change strategies to promote and support physical activity of adequate intensity, duration and type, needed to improve health-related fitness.

**Trial Registration No:**

Australia and New Zealand Clinical Trials Register (ANZCTR): ACTRN12611000976987

## Background

The fitness levels of children and adolescence are in decline [1-3]. This is an alarming trend given that high levels of physical fitness in this age group are associated with improved physical and mental health both in the short -and long-term [4,5]. Recent studies have shown that children who display high levels of physical fitness, especially health-related fitness (HRF) [6], have a decreased risk of developing cardiovascular disease and other chronic illnesses (such as obesity, Type 2 diabetes mellitus, osteoporosis and some cancers) [7], are less likely to suffer from anxiety and depression [8], and more likely to perform better academically [9].

In response to the declining physical activity (PA) and physical fitness (PF) levels of children, and the corresponding increase in non-communicable diseases (NCD), the World Health Organization (WHO) published the *Global Recommendations on Physical Activity and Health *[10]. These recommendations address the link between the frequency, duration, intensity, type and total amount of physical activity needed for preventing NCD [10]. The WHO recommendations now outline that children aged 6-17 years should participate in at least 60 min of moderate-to-vigorous PA every day, and to perform vigorous PA (high intensity), muscle-strengthening PAs and bone-strengthening PAs, on at least three days per week [10]. As such, studies investigating and targeting children's health may also benefit from a redirected focus on regular vigorous intensity PA and improvements in HRF to improve overall health.

A recent review confirms that there is great public health potential for school-based interventions to improve the PA and PF levels of young people [11]. The school, via the curriculum, school ethos and community, is an ideal avenue for accessing and educating young people about the importance of PA, the value of achieving and/or maintaining HRF standards and for building the skills necessary for long-term behaviour change [12]. There are numerous opportunities in the school setting for the promotion of PA, including health and physical education (HPE), active transportation, active breaks, sport etc. While HPE is widely acknowledged the cornerstone of a schools' physical activity program, studies have questioned the quality and quantity of HPE lessons delivered in primary schools [13-15].

Recent studies have demonstrated positive results in improving HRF, especially cardio-respiratory fitness, via school- based interventions [11]. However, many have failed to address the multiple components that influence behaviour in the school setting, make reference to credible learning theories or curriculum direction in intervention designs, or specifically target improvements in all of the HRF components [16]. In addition, few studies have designed and tested multi-component programs to extend learning into the school playground and the home - potentially limiting the impact that the program has on health outcomes and behaviour change [16].

The Fit-4-Fun program is an innovative and engaging school-based fitness education program. It encompasses all of the components of a Health Promoting School [17], extends learning beyond the classroom and provides professionally designed curriculum resources for primary school teachers This study builds upon the Fit-4-Fun pilot study (conducted in 2010) and will provide further evidence to support the effectiveness of the Fit4Fun intervention for improving the HRF and PA levels of children, along with their attitudes towards physical fitness. This paper provides the rationale and study protocol of the Fit-4-Fun program.

## Methods/Design

### Study design

Fit-4-Fun is an 8-week multi-component school-based HRF education intervention and will be evaluated using a group randomized controlled trial (RCT) with 6-month follow-up. Ethics approval for the study was obtained from the University of Newcastle, NSW, Australia and the Newcastle-Maitland Catholic Schools Office, and is registered with the Australian and New Zealand Clinical Trials Registry (ACTRN12611000976987).

Following the initial recruitment process, all eligible participants will complete baseline assessments and follow-up measures will be conducted immediate post-intervention and at 6-months. The design, conduct and reporting of the Fit-4-Fun intervention will adhere to the Consolidation Standards of Reporting Trials (CONSORT) guidelines [18]. School Principals, teachers, parents and study participants will provide written informed consent.

### Recruitment & study participants

**Figure 1:**
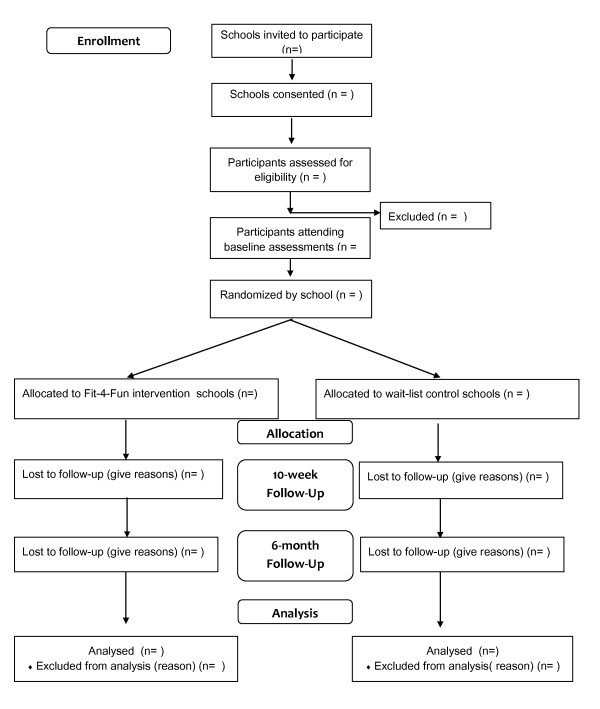
**Flow of participants through the Fit-4-Fun**.

Primary schools from the Hunter Region in NSW, Australia, will be invited to participate in the Fit-4-Fun program in 2011. Initially, school Principals will be contacted via email and then a face-to-face meeting will be arranged. Written consent will be sought from both the Principal and the classroom teachers of each school before participants from Stage 3 classes (years 5 and 6) are recruited. All students are eligible to participate in the program if they return a signed informed consent letter from their parent(s) with child assent, and do not currently have a medical condition or physical injury preventing testing or training. Figure 1 depicts the flow of participants through the trial.

### Sample Size Calculation

A battery of fitness assessments will be conducted to determine the HRF levels of participants. The primary outcome variable in this study is cardio-respiratory fitness. A power calculation was conducted to determine the sample size necessary to detect changes in cardio-respiratory fitness (VO2max). Based on a previous study by Kolle et.al (2009), an increase of 6 mL/kg/min was regarded as clinically important and achievable in children [19]. Using an alpha of 0.05 and power of 80%, a sample size of 128 will be needed to detect a 6 mL/kg/min difference between groups. To account for the clustered nature of the data and participant drop-out, we will aim to recruit 200 participants from four schools.

### Blinding & Randomization

Participants and research assistants will be blinded to treatment conditions during baseline assessments. Randomization by school will be performed at the completion of baseline assessments and the four participating schools will be randomly assigned to the Fit-4-Fun intervention (2 schools) or a 9-month wait-list control group (2 schools). A randomization envelope will be prepared by a member of the research team and an independent third party will blindly allocate the four schools into one of the two treatment conditions.

### Training

Research assistants will conduct and record all physiological assessments, and will administer the student questionnaire. All research assistants will complete an identical training session prior to assessments to maintain consistency and where possible the same assessors will be used for all assessments.

### Treatments

#### a) Intervention

##### Theoretical framework

The Fit-4-Fun Program is grounded in Bandura's Social Cognitive Theory and Harter's Competence Motivation Theory and aims to provide children with the knowledge and skills necessary for short- and long-term behaviour change [20,21]. The program also aims to promote the development and maintenance of positive PA behaviours and attitudes among participants, by targeting possible mediators of behaviour change (e.g. social support, self-efficacy, supportive environment, enjoyment) [21,22]. An overview of the Fit-4-Fun program content and alignment with theoretical constructs is reported in Table 1.

**Table 1:** 'Fit-4-Fun' program content and alignment with theoretical constructs

Wk	Session focus	Session overview	Behaviour change strategies	SCT/CMT construct
**1**	Health-related fitness (theory)	• Programme rational • Defining PA & PF • HRF & SRF • PA guidelines • Analysing current PA & PF behaviours • Overview of 'Home Activity Programme'	• Provide information about PA & PF behaviours/link to health • Develop self-monitoring skills (weekly PA timetable, talk test) • Provide social support and encouragement (to meet PA guidelines) • Develop goal setting skills (HW task)	• Outcome expectations • Social support (home & school) • Self-efficacy • Intentions • Motivation

**2**	Cardio-respiratory fitness (CRF) (theory)	• Provide information on CRF • Role of heart & lungs during PA • Linking heart rate (HR) to PA intensity (lab) • Linking CRF & health	• Provide information about CRF & the role of the heart & lungs during PA • Develop skills in self-monitoring (using heart rate) • Predicting consequences of actions • Making recommendations relating to PA and CF	• Outcome expectations • Self-efficacy • Social support • Motivation

**3**	Improving cardio-respiratory fitness (practical)	• Revise CRF & measuring intensity using HR • Participate in a practical PE lesson with a gross motor warm-up activity, dynamic stretches, skill development activities, modified games and cool-down • HR is monitored throughout the lesson • Discussion about the type of PA and heart rate (high intensity/vigorous)	• Provide opportunity to participate in enjoyable physical activities in a supportive environment • Maximal participation is provided for and encouraged • Positive feedback is provided throughout the session • Students are to reflect on their performance and re-assess current PA behaviours	• Outcome expectations • Social support • Self-efficacy • Motivation

**4**	Muscular Fitness (MF) (theory)	• Define MF • Muscular strength Vs Muscular endurance • Activities that require MF • Measuring MF (lab) • Linking MF & health • Improving MF	• Provide information on MF • Link current PA behaviour to MF • Develop goal setting skills/set targets to achieve • Self-monitoring skills (PF tests) • Participation in non-threatening practical assessments (enjoyment)	• Outcome expectations • Social support • Self-efficacy • Intentions • Motivation

**5**	Improving muscular fitness (practical)	• Revise MF & measuring MF • Participate in a practical PE lesson with a gross motor warm-up activity, dynamic stretches, MF circuit and cool-down • HR is monitored throughout the lesson • Discussion about the type of PA and MF (resistance)	• Provide opportunity to participate in enjoyable physical activities in a supportive environment • Maximal participation is provided for and encouraged • Positive feedback is provided throughout the session • Students are to reflect on their performance and re-assess current PA behaviours	• Outcome expectations • Social support • Self-efficacy • Motivation

**6**	Flexibility (theory)	• Define flexibility • Activities that require MF • Benefits of being flexible • Types of stretching • Improving flexibility (lab) • Linking MF & health • Improving MF • Predicting outcomes from changed MF behaviours • Goal setting task • Link flexibility to lifestyle behaviours	• Provide information on flexibility • Link current PA behaviour to flexibility • Develop goal setting skills/set targets to achieve • Self-monitoring skills (PF tests) • Participation in non-threatening practical assessments (enjoyment)	• Outcome expectations • Social support • Self-efficacy • Intentions • Motivation

**7**	Improving flexibility (practical)	• Revise flexibility and measuring flexibility • Participate in a practical PE lesson with a gross motor warm-up activity, dynamic stretches, fun stretching routines and cool-down • HR is monitored throughout the lesson • Discussion about the type of PA and improved flexibility	• Provide opportunity to participate in enjoyable physical activities in a supportive environment • Maximal participation is provided for and encouraged • Positive feedback is provided throughout the session • Students are to reflect on their performance and re-assess current PA behaviours • Link to lifelong behaviours	• Outcome expectations • Social support • Self-efficacy • Motivation

**8**	Improving health-related fitness through games (practical)	• Revise HRF components • Revise improving HRF • Participate in a student-centred practical PE lesson where students adapt fun games to incorporate HRF • HR is monitored throughout the lesson • Discussion about the type of PA and improved HRF • Summary of health benefits with improved HRF • Evaluation of 'Fit-4-Fun'	• Provide opportunity to participate in enjoyable physical activities in a supportive environment • Maximal participation is provided for and encouraged • Positive feedback is provided throughout the session • Students learn skills in adapting PA to improve HRF • Students are to reflect on their performance and re-assess current PA behaviours • Link to lifelong behaviours	• Outcome expectations • Self-efficacy • Social Support • Motivation

**1-8**	'Fit-4-Fun' Home Activities	• Participation in an 8 week home activity programme • 3 weekdays: MF, flexibility, CRF activities • 1 weekday: fitness assessments • Weekends: family activities & CRF assessment • Weeks 1, 5, 8: Goal setting tasks • Problem Solving Task (assessment)	• Students participate in a range of fun activities with their parents/siblings • Family provide social support throughout the programme • Students develop skills in self-monitoring and self-motivating • Students develop skills in goal setting & time management • Students develop skills in assessing & planning to improve the physical environment	• Outcome expectations • Self-efficacy • Social Support • Motivation

Abbreviations: SCT- Social Cognitive Theory; CMT - Competence Motivation Theory; HRF - Health-Related Fitness; HR - Heart rate;

CRF - Cardio-respiratory fitness; MF - Muscular fitness; PA- Physical activity

The Fit-4-Fun Program includes three major components that are based on the HPS Framework [17]:

##### Curriculum program

An 8-week × 60-min HPE program based on the NSW K-6 syllabus [23] will be delivered during normal HPE lesson time [23]. The program is designed to improve the knowledge, skills and understanding of students in relation to HRF and also focuses on developing skills in assessing and monitoring HRF components. The program overview has been summarized in Table 1. The Fit-4-Fun program will be delivered by a member of the research team who is an experienced physical educator.

##### Family partnership

Children, their parents and family members will be provided with an 8-week home activity program designed to improve HRF levels using a range of engaging and enjoyable fitness activities, small-sided games and fitness challenges (3 × 20 min per week for 8 weeks). Children will select from a wide range of activities that are specifically designed to improve muscular fitness, flexibility and cardio-respiratory fitness. There are also goal setting activities and reflection tasks for students to complete with their parents throughout the program, enabling them to set personal fitness goals, monitor their achievement and to reflect on their progress.

##### School environment

Schools will be provided with activity task cards outlining the rules and organization of a range of fun and vigorous games (e.g. small-sided invasion games, skipping challenges) and a variety of equipment for use during break-times. The student directed break-time activities will involve participation in enjoyable games, activities and fitness challenges.

Social support for participation in all program activities will be provided by teachers, parents, and students throughout the intervention period. For example, teachers will verbally encourage students to join in the break-time games, there will be posters pinned at the exit points of the classroom reminding students to complete their home activities and to be active at lunch and recess, Fit-4-Fun leaders (students) will be asked to encourage other students to participate in activities and to make the equipment available for use, and parents will be asked to support and encourage their child at home. In addition, notices will be placed in the school newsletter and local media supporting the program and an incentive/award scheme for student participation will be in place. Students who complete home tasks and participate during curriculum sessions will be eligible to receive a gold, silver or bronze award.

The strategies used in the Fit-4-Fun program to target mediators of behaviour change are as follows:

##### Enjoyment

Many authors have argued that "fun" or enjoyment is considered one of the most important reasons that children and adolescents become involved and to continue to participate in physical activity - and a lack of fun or enjoyment is likely to lead them to withdraw [24,25]. Therefore, all of the programs components will involve participation in 'fun' and engaging physical activities, games, challenges or learning activities that children enjoy.

##### Self-efficacy

Self-efficacy is the central determinant of health behaviour change in SCT as self-efficacy beliefs directly and in-directly influence motivation, affect and behaviour [22,26]. Data suggest that there is a positive correlation between self-efficacy and the amount of vigorous physical activity in children and youth [27-29]. The techniques that are used in the Fit-4-Fun program and that have been shown to significantly improve physical activity self-efficacy, and therefore physical activity behaviours, include goal setting/action planning, positive reinforcement for effort or progress towards a set behaviour, the provision of instruction and feedback on performance, self-monitoring, self- regulation, the provision of information on consequences of behaviour and skills practice [30].

##### Supportive Environment

The school's social and physical environments are related to the facilitation or constraint of child and adolescent physical activity [31-34]. Strategies that have been implemented in the Fit-4-Fun program to improve the school and home environment include: increased access to play and sports equipment, provision of quality physical education lessons, and on-going positive reinforcement and *social support *from parents, teachers and peers [35-37].

#### Control (wait- list control group)

The control group will participate in their usual 60 min/week HPE lesson over the 8-week intervention period and will be delivered by their normal classroom teacher. The lesson content will be determined by the set school HPE program. The control group will receive the Fit-4-Fun program resources at the completion of the study period.

### Outcome measures

Demographic information (i.e., age, sex, language spoken at home, country of birth) and physical fitness cognitions (i.e., enjoyment, perceived social support, perceived environmental support, physical activity self-efficacy) will be collected via a questionnaire, and physiological data will be collected using the measures detailed below.

A battery of HRF field-based assessments will be conducted one week prior to intervention commencement. Field-based tests will be used as they provide an alternative to laboratory tests, since they are time efficient, cheaper, require fewer resources and can accommodate for multiple participants at once [38]. The testing environment will be identical for both baseline and follow-up measures and all tests, other than the beep test, will be performed in groups of three or four students with a trained research assistant remaining with the group for all assessments. The physiological fitness tests include:

#### Cardio-respiratory fitness (CRF)

##### 20 m shuttle run test (Beep test)

The participant will be required to run back and forth between two lines, 20 m apart, within a set time limit. Running speed will commence at 8.5 km/hr and will increase by 0.5 km/hr each minute using the 20 m Shuttle Run Test cadence CD. Participants will be instructed to run in a straight line, to place one foot over the 20 m line and to pace themselves according to the audio CD. The test requires maximal effort and participants are required to run until they can no longer keep up with the speed set by the tape. The level and number of shuttles within the level completed will be recorded [39].

#### Flexibility

##### Sit and reach test

Using standardized protocols as detailed in the FITNESSGRAM/ACTIVITYGRAM Reference Guide [40] the participant will perform the sit and reach test on the right leg, the left leg and both legs together. Double leg scores, followed by single leg (back saver) measures will be recorded in centimetres. A negative score on the sit and reach test indicates that the participant does not reach the level of the toes and a positive score indicates that the participant reaches beyond the level of the toes.

#### Muscular fitness (MF)

##### Standing jump [41,42]

The participant will be required to stand with both feet parallel and behind a marked starting line. The participant will be asked to swing their arms backwards and then forwards and to jump with both feet simultaneously as far forward as possible. Two attempts at the jump will be permitted with the furthest jump being recorded in meters. The distance measured is the distance between the starting line and the closest landing position (back of the heel).

##### 7- stage sit-up test [43]

The participant will lie on their back, with their knees at right angles and feet flat on the floor. The participant then attempts to perform one complete sit-up for each level in the manner prescribed below, starting at level 1. Each level is achieved if a single sit up is performed in the prescribed manner, without the feet coming off the floor. A second attempt is permitted if a level is not reached. The highest level sit-up correctly completed is recorded.

Level and Description:

0 = cannot perform level 1

1 = with arms extended, the athlete curls up so that the wrists reach the knees

2 = with arms extended, the athlete curls up so that the elbows reach the knees

3 = with the arms held together across abdominals, the athletes curls up so that the chest touches the thighs

4 = with the arms held across chest, holding the opposite shoulders, the athlete curls up so that the forearms touch the thighs

5 = with the hands held behind head, the athlete curls up so that the chest touches the thighs

6 = as per level 5, with a 5 lb (2.5 kg) weight held behind head, chest touching the thighs

7 = as per level 5, with a 10 lb (5 kg) weight held behind head, chest touching the thighs

##### Basketball throw test [44]

The participant sits on the floor with their buttocks, back, shoulders and head remaining against the wall and their legs straight with feet together. An assistant places a hoop on top of the participant's toes and the participant assumes the chest pass position with elbows touching the wall. The participant will perform a two-handed chest pass through the hoop and the distance from the wall to the ball's first point of contact on the ground is measured in metres (m). Each participant performs two trials.

##### Push-up test [40]

The participant will start in push-up position with their hands shoulder width apart and directly below their shoulders. Keeping the back and knees straight, the participant will lower the body until there is a 90-degree angle at the elbows, with the upper arms parallel to the floor, and then they will push back up to full extension of the arms. The push-ups will be performed in time to a metronome set at 40 beats per minute (bpm) and the participant will push-up on one beat and down on the next (20 push-ups per minute). The participant will continue until they can do no more in rhythm. The number of complete push-ups performed will be recorded.

#### Body composition

##### Height [44]

Height will be measured without shoes to the nearest 0.1 cm using the stretch stature method on a portable stadiometer (model no. PE087, Mentone Educational Centre, Australia). Height will be measures twice, with accepted values within 0.3 cm. A third measure will be taken if measures are not within the accepted range. The average of two acceptable measures will be reported.

##### Weight [44]

Weight will be measured to the nearest 0.1 kg in light clothing and without shoes using calibrated digital scales (Model no. UC-321PC, A&D Company Ltd, Tokyo Japan). Weight will be measures twice, with accepted values within 0.1 kg. A third measure will be taken if measures are not within the accepted range. The average of two acceptable measures will be reported.

##### Body Mass Index (BMI) [44]

BMI will be calculated using the formulae BMI = mass (kg)/height (m).2. Body mass index z-scores (BMIz) (measures of relative weight adjusted for child's age and sex) [45] will also be used to determine relative weight status based on international data [46].

#### Physical activity (PA)

The participant will be asked to wear a sealed Yamax SW700 pedometers (Yamax Corporation, Kumamoto City, Japan) during their normal daily activities to measure PA for seven days (including three consecutive days and one weekend day) [47]. This is a validated objective measure of physical activity for use with young people [48]. The participants will be asked to wear the pedometers at all times other than when sleeping or when they might get wet. Classroom teachers will record the step counts and then reset the pedometers of participants at the start of the school day (9 am) on Monday through to Friday during the assessment periods. On the weekend parents will be asked to record the step count readings of their child and to reset the pedometer as close to 9 am as possible. Any problems with recordings or participation in water-based activities are to be noted on the recording sheet and non-ambulatory activities are to be adjusted for on the daily step count via imputation. If imputation is required then a total of 1000 steps for 10 minutes of moderate -vigorous activity and 1500 steps for vigorous activity will be added to the participants step counts for the given time period [49].

#### Physical fitness testing experience and attitudes towards physical fitness testing

##### Student Questionnaire

The 'Fit 4 Fun' Student Questionnaire will be administered to participants at baseline, immediate post-intervention and 6-month follow-up and has been designed to collect information about the attitudes, opinions, behaviours and characteristics of the children involved in the Fit-4-Fun research project. The questionnaire design and purpose is described below.

*Demographic Information*: Six structured quick response questions will be used to determine the personal characteristics of the children participating in the study (age, DOB, school year, language, country of birth).

*Fitness testing experience*: Information relating to the participant's experience with fitness testing is sought through the use of five structured closed and semi-closed questions.

*Self-efficacy*: Information relating to participant's self-efficacy for PA will be measured using eight questions. The scale uses a single factor 5-point Likert format and is an adapted version of an 8-item questionnaire previously developed for use with 5th, 8th and 9th grade girls (PASES) [50-52]. The child is asked to select how much they agree with the eight statements by ticking the relevant circle ("Disagree a lot" though to "Agree a lot"). Each item is scored from 1 to 5, where a score of 1 indicates low self-efficacy. E.g. "I can be physically active even if it is hot or cold outside".

*Enjoyment*: of physical activity will be assessed through six negatively worded questions. The scale uses a 5-point Likert format and is an adapted version of the a 16-item version of the Physical Activity Enjoyment Scale (PACES) [53] and has been recently validated for use with children [54-56]. The child is asked to select how often they experience the relevant feeling about physical activity by ticking the relevant circle ("Never" though to "Every day"). Each item is scored from 1 to 5, where a score of 1 indicates low levels of enjoyment. E.g. When I am physically active........... It's no fun at all.

*Social Support for PA*: Children are required to indicate the level of social support for physical activity they receive from friends, family and teachers. The three scales use a 5-point Likert format and have been adapted from two scales used in the student survey of the Amherst Health and Activity Study [57]. Responses are sought for 3-items pertaining to social support from friends, 4 for social support from family and 4-items relating to social support provided by teachers. The structured scales use a 5-point Likert format and have been recently tested for validity and use with children by Dishman and colleagues (family and friend scales only) [55]. The teacher social support scale has been devised for the purpose of this study and follows the structure and wording of the other two scales. Children are asked to select how often a specific form of social support is provided to them during a typical week by ticking the relevant circle ("Never" though to "Every day"). Each item is scored from 1 to 5, where a score of 1 indicates low levels of social support. Scores are summed and then averaged, resulting in a scale mean. E.g. "During a typical week at school, how often do your FRIENDS.... do physical activity or play sports with you?"

*Perception of the School Physical Environment*: Information relating to the physical environment of the school is sought through 8 structured questions in part E of the questionnaire. The scale uses a single factor 4-point Likert format and is an adapted version of the 2-factor, 20-item questionnaire Q-SPACE developed by Robertson-Wilson, Levesque & Holden (2007). The child is asked to select how much they agree with the eight statements by ticking the relevant circle ("Strongly Disagree" though to "Strongly Agree"). Each item is scored from 1 to 4, where a score of 1 indicates a low level of support for physical activity in the school's physical environment (e.g. availability of equipment, play areas, supervision).

E.g. "There is sports equipment available for students to use during recess and lunch breaks"

*The School Environment Audit*: An audit will be completed by two independent research assistants to evaluate the school environment and its relationship to physical activity. The audit will use a purpose designed scale based on The School Environment Audit Tool [58] and the Physical Activity School Scan (PASS) [59]. The assessor will be asked to rate the quality and quantity of specific physical components of the school environment, including sport and play facilities, surrounding bike paths, playground design, aesthetics and sports equipment.

#### Process evaluation

The feasibility of the program will be examined using a number of strategies. Measures of recruitment (i.e., evaluation of the recruitment process, dissemination of information and obtaining informed consent), retention (i.e., how many students completed the program and participated in all assessments pre and post-intervention), adherence (i.e., the degree to which staff and students followed the Fit-4-Fun program), and satisfaction (i.e., level of satisfaction and engagement in the program by students, staff and parents) will be used. Evaluation questionnaires will also be administered to determine students' and teachers' perceptions of the various program components, attendance, and participation in extra-curricular activities. A 6-point Likert scale format will be used with responses ranging from "Strongly Disagree" through to "Strongly Agree" (e.g. "I think all schools should have the Fit-4-Fun Program"). Focus group interviews involving 2-3 students and lasting 5-10 minutes will also be conducted by trained research assistants to examine the perceptions of students about the Fit-4-Fun program. The groups will be based on friendship groups (both single-sex and mixed-sex groups) and will utilize standardized semi-structured questions. The anonymous verbal responses will be recorded by the research assistant. The following questions will be asked: What did you like about the Fit-4-Funprogram? What didn't you like about the Fit-4-Fun program? Did your activity levels change during the breaks at school/how? Were your parents/family interested/engaged in the home activities/how? How have your skills/attitudes/behaviours towards physical fitness changed over the past 8 weeks? How? What changes would you make to improve the program in the future? At the end of the session the participants will also be asked if they have anything else to add or would like to discuss anything further.

### Statistical Methods

Statistical analyses will be conducted using linear mixed models with PROC MIXED in SAS V 9.1 (SAS Institute Inc, Cary, NC) and alpha levels will be set at *p *< .05. The models will be specified to adjust for the clustered nature of the data and multiple imputations will be considered if the dropout rate is substantial. Differences between participants in the intervention and groups at baseline and differences between completers and those who drop out of the study will be examined using Chi square and independent samples t-tests in PASW Statistics 17 (SPSS Inc. Chicago, IL) software.

Focus group responses will be analysed using an inductive analysis where an initial exploration of the verbal responses will be used to identify any patterns or themes [60]. Using a recursive approach, quotes with similar meanings will be grouped together and labelled with a 'theme' [61]. A concept map will then be created to give a visual display of the themes and to aid in providing an accurate description and interpretation of the focus group data.

## Discussion

The Fit-4-Fun study described in this paper is one of the first RCTs in Australia to specifically target the HRF levels of primary school children. The results of this study will provide further evidence to support the feasibility and efficacy of the Fit-4-Fun intervention for improving the HRF and PA levels of children, along with their attitudes towards physical fitness.

This study addresses some of the limitations found in previous interventions by: (1) specifically targeting all of the components of HRF in primary school children; (2) taking a multi-faceted approach to facilitating behaviour change via the HPS Framework; (3) having a theoretically- and curriculum-based program; (4) extending HRF education beyond the classroom and into the home; and (5) by using enjoyable and engaging learning activities to motivate students to adopt healthy behaviours.

The findings of this study will provide valuable information for other research groups looking to improve the HRF levels of children via school-based interventions. Furthermore, it will ascertain whether the Fit-4-Fun program is an effective program for future large-scale implementation.

## Competing interests

The authors declare that they have no competing interests.

## Authors' contributions

NE, PJM, and DRL obtained funding for the research. All authors contributed to developing the protocols and reviewing, editing, and approving the final version of the paper. NE is the guarantor and accepts full responsibility for the conduct of the study. All authors have read and approved the final manuscript.

## Pre-publication history

The pre-publication history for this paper can be accessed here:

http://www.biomedcentral.com/1471-2458/11/902/prepub
